# Synesthesia and learning: a critical review and novel theory

**DOI:** 10.3389/fnhum.2014.00098

**Published:** 2014-02-28

**Authors:** Marcus R. Watson, Kathleen A. Akins, Chris Spiker, Lyle Crawford, James T. Enns

**Affiliations:** ^1^Department of Ophthalmology and Visual Sciences, Child and Family Research Institute, University of British ColumbiaVancouver, BC, Canada; ^2^Department of Philosophy, Simon Fraser UniversityBurnaby, BC, Canada; ^3^Department of Psychology, University of British ColumbiaVancouver, BC, Canada

**Keywords:** synesthesia, learning and memory, plasticity and learning, perceptual development, cognitive development, multisensory processing

## Abstract

Learning and synesthesia are profoundly interconnected. On the one hand, the development of synesthesia is clearly influenced by learning. Synesthetic inducers – the stimuli that evoke these unusual experiences – often involve the perception of complex properties learned in early childhood, e.g., letters, musical notes, numbers, months of the year, and even swimming strokes. Further, recent research has shown that the associations individual synesthetes make with these learned inducers are not arbitrary, but are strongly influenced by the structure of the learned domain. For instance, the synesthetic colors of letters are partially determined by letter frequency and the relative positions of letters in the alphabet. On the other hand, there is also a small, but growing, body of literature which shows that synesthesia can influence or be helpful in learning. For instance, synesthetes appear to be able to use their unusual experiences as mnemonic devices and can even exploit them while learning novel abstract categories. Here we review these two directions of influence and argue that they are interconnected. We propose that synesthesia arises, at least in part, because of the cognitive demands of learning in childhood, and that it is used to aid perception and understanding of a variety of learned categories. Our thesis is that the structural similarities between synesthetic triggering stimuli and synesthetic experiences are the remnants, the fossilized traces, of past learning challenges for which synsethesia was helpful.

## INTRODUCTION

When synesthesia first came under scientific scrutiny in the 1800s, a fairly common view was that synesthetic experiences were learned, and moreover served to help synesthetes with tasks such as mathematics, remembering sequences, and many other learning challenges (e.g., Galton, 1881; Calkins, 1893; Jewanski et al., 2011). After being largely ignored for much of the twentieth century, synesthesia returned to the scientific mainstream about 25 years ago but learning was largely rejected as either a cause or function of synesthesia. For example, Cytowic (1989), who virtually single-handedly re-introduced synesthesia to the modern research community, originally argued that “synesthesia is not learned” and many other researchers were similarly at pains to distinguish learned or remembered associations from “real” synesthesia (cf. Harrison and Baron-Cohen, 1996; Elias et al., 2003; Ramachandran and Hubbard, 2003).

In recent years, however, it has become clear that the earlier viewpoint needs to be taken seriously. There has been a flurry of research on the profound impact of learning on synesthesia, and several prominent researchers now argue that learning and conceptual factors are critical components of synesthetic development (cf. Simner et al., 2009a; Jürgens and Nikolic, 2012; Deroy and Spence, 2013a; Witthoft and Winawer, 2013). And some of those who formerly denied the role of learning appear to have reversed their stance (cf., Cytowic and Eagleman, 2009). There has also been a smaller body of literature dealing with influences in the other direction: the utility of synesthesia for learning new material.

These two streams of research are the focus of the first two sections of this review. In the final section, we present a theory of synesthetic development that is motivated by our interpretation and integration of the literature in both streams. Here we argue that synesthesia may develop in part as a strategy for coping with the learning demands of childhood, and that the successful application of this strategy molds synesthetic associations to reflect what has been learned. At the outset, we wish to note that this need not be in conflict with genetic or nativist accounts of synesthesia. There may be a particular genetic inheritance or neurological profile that is required for the development of synesthesia, but learning is also a crucial component of this development, as is explicitly acknowledged by researchers with a strongly nativist approach (e.g., Spector and Maurer, 2009). The theory presented here goes further by stating that synesthetic associations are not merely learned, but learned *for strategic purposes*. But strategic purposes do not rule out an equally important role for genetic or neurological factors.

In what follows, we use the standard terminology of “inducer” to refer to the stimuli that trigger synesthetic experiences, and “concurrent” to refer to these experiences themselves, and we generally refer to types of synesthesia by the common formula *inducer–concurrent *(e.g., *time–space *synesthesia refers to time being perceived by the synesthete in a spatial form).

## HOW LEARNING INFLUENCES SYNESTHESIA

Synesthesia typically develops during a period in which children are engaged in the explicit learning of new skills. This often occurs in a formal setting, and involves learning to recognize, discriminate, name, and use the elements of highly structured categories. These may be the letters of the alphabet, the months of the year, the keys of music – the synesthetic inducers themselves. This learning phase is often very slow and deliberate, continuing into the teenage years. As the child learns more about the inducer domain, their synesthetic experiences change. Not only are individual concurrents determined on the basis of this learning, but so too are the relations *between *concurrents. Indeed synesthetic concurrents can be said to *encode *a wide range of information about the inducers, albeit in a highly idiosyncratic manner unique to each synesthete. We justify each of these claims below.

### SYNESTHETIC INDUCERS ARE LEARNED CATEGORIES

A striking feature of almost all cases of synesthesia is that they presuppose complex categorical learning. They involve a consistent, one-to-one mapping of members of the inducer class onto members of the concurrent class, which requires the ability to identify and discriminate the members of the inducer class and, more generally, to understand the nature of that class. A synesthete who sees each *L *as lime green can visually identify that letter by means of its shape. But the synesthete also sees *L as *a letter: a symbol that represents specific phonemes in the context of words, and a member of an ordered sequence, among other things. If an instrument–color synesthete “hears” a piccolo and a flute as two distinct shades of blue, the synesthete must be able to discriminate between the characteristic timbres of the two instruments, but also to classify and recognize them as distinctive timbres. Such understanding does not come easily.

The development of literacy and letter recognition is a notorious example. Even 11 year olds, who have had a minimum of 6 years of literacy training, are affected by letter crowding over larger expanses of text (7.13 threshold keystrokes) than are adults (2.83 keystrokes). In fact, crowding distance is roughly the same for 11 year olds as it is for 8 and 5 year olds (Jeon et al., 2010). Sequential and cyclical systems for marking the passage of time, both natural (e.g., day and night, seasons) and conventional (e.g., days of the week, months) present their own conceptual hurdles. For example, while the child’s understanding of sequential order and mistakes in sequential order improves progressively from ages 4 to 11, only at age 10 do children begin to form an integrated understanding of two distinct cyclical orders of events, allowing them to order the months of the year and recognize when standard holidays are located (Friedman, 1977). Similarly, musical categories and relations such as absolute and relative pitch, scales, modes and tunings, and the timbre of specific instruments, all require either extended listening experience and/or explicit musical instruction. These three classes, graphemes, time units, and aspects of music, make up most reported cases of synesthetic inducers in all large-scale studies to date (cf. Day, 2005; Rich et al., 2005; Simner et al., 2006), and, like less common inducers such as swimming styles (Nikolic et al., 2011) are typically learned only with considerable effort over long periods of time as a result of explicit instruction from teachers or parents.

What about the rarer types of synesthesia that involve “natural” inducers such as odors, sounds, or pains (cf. Day, 2005)? Neonatal behavior such as crying presumably reflects the experiences of the newborn, such as states of hunger, pain, thermal discomfort, or agitation. But despite the presence of such experiences, the perceptual and conceptual skills that undergird developmental synesthesia, that make possible mappings between inducer and concurrent domains, are arguably not present at birth. In fact, what unifies many of these inducers is that (a) they are notoriously difficult to identify and name, (b) their identification and naming is aided by contextual cues and hence these senses are highly multimodal, and (c) discrimination is affected by the naming. To use the example of odor, both odor naming and discrimination have an inverted U-shaped function across age, with young children and the elderly least able to name and discriminate familiar odors (de Wijk and Cain, 1994a, b). Even for young adults, odor naming is very difficult. In blind testing without visual or other cues, adults recognized only 50% of common household odors. The percentage is much lower when less common odors are tested. However, in a phenomenon called “tip of the nose” effect, Jönsson et al. (2005) have shown that providing the name immediately serves to “clarify” the odor for the perceiver. Similarly, when four names are offered in conjunction with the odor sample, if one name matches the sample, correct identification of odor increases from 30% to 80% (again in young participants; de Wijk and Cain, 1994b). Finally, what people smell is strongly influenced by color, and these associations between color and odor are at least partly culturally determined. For instance, Shankar et al. (2010) found that when presented with the same brown colored liquid, 70% of the British participants identified the drink as a cola while none of the Taiwanese participants did so. Instead, 49% of the Taiwanese identified that drink as grape.

Now consider the example of pain–color synesthesia. The few studies that exist suggest that *categories *or *types *of pain are the inducers, e.g., headaches and cramps reliably induce very different concurrents (Coriat, 1913; Dudycha and Dudycha, 1935). But reliably categorizing pain requires learning. At birth, a cold and hungry baby will cry. But electrophysiological recordings suggest that young infants cannot distinguish a soft touch on the heel from a noxious lancing (Fabrizi et al., 2011). Even older children (age 4–11) with a great deal of experience with pain often identify loss of appetite or nausea as pain (Kortesluoma and Nikkonen, 2004). The number and type of pains reported by children is influenced by their own past experience with pain, and by the pains they have observed their parents and siblings experiencing (Harbeck and Peterson, 1992; Franck et al., 2010), and the terms used to describe different types of pain become increasingly more specific through childhood (e.g., Gaffney and Dunne, 1986; Harbeck and Peterson, 1992; Crow, 1997). Moreover, children’s judgments of the severity of pain are mediated by cues such as syringes, or their own crying or sweating (Kortesluoma and Nikkonen, 2004). All this evidence suggests that a large part of the ability to categorize the quality, magnitude, and location of pains is improved and refined throughout childhood.

These examples suggest that the apparent dichotomy between “innate” and “learned” varieties of synesthesia needs closer scrutiny. Certainly, nothing logically precludes innate sensory representations or categorizations. But learning may influence the categorization of *all *synesthetic inducers, not merely the large majority of cases that clearly involve extensive explicit category learning.

In summary, s*ynesthesia normally develops as part of a difficult, formal process of learning to recognize and categorize perceptually and conceptually complex inducers. Even in cases of more unusual inducers, learning – especially perceptually and/or conceptually challenging forms of learning – seems to be at the heart of synesthesia.*

### THE SLOW COURSE OF SYNESTHETIC DEVELOPMENT

Only two published papers directly investigate the development of synesthesia, both derived from the same longitudinal database on grapheme–color associations (Simner et al., 2009a; Simner and Bain, 2013). These show that synesthetic associations coalesce very slowly: 6- to 7-year-old synesthetes have consistent color associations with approximately 35% of letters, which increases to approximately 70% by ages 10–11. Thus there is a period of at least 4 years during which these synesthetic associations are in flux. Unfortunately the published accounts do not tell us about those letters that do not have stable associations with colors. Do they have weak associations, unstable associations, or no associations at all? We anticipate that future child studies will attempt to grapple with this issue.

Interestingly, not a single one of Simner et al. (2009a), Simner and Bain (2013) 615 participants had perfectly stable letter–color associations, a fact that should make us question the common claim of synesthetes that letter colors are fixed “from infancy” or “as long as I can remember” (e.g., Rich et al., 2005). It appears that the color evoked by a letter in adulthood is experienced as the color that *L *has “always been,” but these memories are not always accurate.

Synesthesia often develops slowly, into late childhood.

### LEARNED FIRST-ORDER INFLUENCES ON SYNESTHETIC DEVELOPMENT

While Simner et al. (2009a) have provided the only studies of childhood synesthetes, the settled associations of adult synesthetes provide insight into the multi-year process of gradual synesthetic stabilization. Just as paleontologists can infer a great deal about long-extinct species by examining their remains, synesthesia researchers can learn about various influences on the development of synesthesia by examining the stable associations that persist into adulthood, making these associations like perceptual/cognitive fossils. We divide these associations into two broad groups: first-order and second-order mappings. In principle there could be learned or innate influences of both types, but as we illustrate below, in most cases these influences are clearly learned, and the evidence for the innateness of some cases is debatable.

A first-order mapping is one in which single elements in one domain (e.g., the domain of letters) are mapped to single elements in another (e.g., the domain of colors). Cases in which particular letters tend to be associated with the same color categories for many synesthetes, then, would be first-order mappings. For example, a common finding is that the first letters of common color names tend to be associated with the corresponding color, for example purple is often associated with *P *for English synesthetes (Simner et al., 2005). Similarly, color *words *are generally the color that they refer to (e.g., the English word *blue *is often blue), and the words for objects with prototypical colors are often colored accordingly (e.g., *banana *is yellow) in both English and Japanese (Baron-Cohen et al., 1987; Rich et al., 2005; Asano and Yokosawa, 2012). All of these associations are clearly learned, i.e., based upon semantic or orthographic associations.

More idiosyncratic cases of learned first-order synesthetic associations exist. Some adult grapheme–color synesthetes have colors that derive almost entirely from childhood toys (Hancock, 2006; Witthoft and Winawer, 2006, 2013) or colored letters displayed in kindergarten classrooms (Colizoli et al., 2012). Similar childhood determinants of synesthetic associations have been noted for well over a century (Calkins, 1893). The origins of these associations can be complex and subtle, and can only be unearthed by careful case studies. As an example, Melanie Ahrling gives this account of some of the childhood origins of the colors and form of her synesthetic calendar:

My entire week is composed of elongated rectangles strung together in a slightly irregular fashion – jutting out a little, either at the top or the bottom. The blue Monday and the yellow-red Tuesday are rounded off at the top – similar to an inverted awning. Work-days have more individual characteristics than the weekends. I’m pretty sure that’s because as a child, I had a different lesson plan for every day and also varying lesson times in the afternoons. Even then, I never really liked Thursdays which for me are dark-green with black stripes due to years of having to go to children’s ballet lessons with an ancient and rather “strict” teacher, which wasn’t exactly my idea of fun. My Wednesday however, is always light-blue with little white clouds. This is possibly because throughout my entire childhood we always had Wednesday afternoons off – in comparison to the other weekdays, on which there was always some kind of schedule that had to be adhered to [...]

For the most part, the weekend was filled with enjoyable outings and for as long as I can remember, has been composed of a large block of two rectangles – namely the dark violet-blue Saturday and the violet Sunday. These rectangles were always somewhat bigger than the rest of the weekdays – and still are – to this day. The more rigidly structured the weekend – irrespective of whether the activities are fun or not – the closer these two rectangles are pushed together (Dittmar, 2009, pp. 139–140).

Such recollections are very difficult to confirm, of course, but they are common in self-reports by synesthetes.

Rarer forms of synesthesia, such as the lexical–gustatory variety, also exhibit learned first-order influences on synesthetic concurrents. Thus the flavor of a word is often influenced by shared phonology between the word and the name of the flavor, so *Cincinatti *might taste of *cinammon*, or by semantic associations between the word and the flavor, so *blue *might taste *inky *(Ward and Simner, 2003; Ward et al., 2005). Even without formal instruction in flavor recognition, semantic associations with tastes still develop.

For grapheme–color synesthetes, *O*, *I*, *X*, and *Z *are typically associated with achromatic colors, in particular *O *and *I *with white and *X *and *Z *with black, and *C *is often associated with yellow (Rich et al., 2005; Simner et al., 2005). Spector and Maurer (2008), 2009, (2011) investigated these associations with two-alternative forced-choice tasks in which participants searched for plastic shapes in containers that were divided into two differently colored halves. They found that non-synesthetic adults, children, and pre-literate toddlers reliably chose to look into the white half of containers when searching for *O *or *I *shapes, the black half of containers when searching for *X *or *Z *shapes, and the yellow half of containers when searching for *C *shapes. These age groups differed, however, in other cases: older children and adults looked into the blue half of containers to find *B* shapes, the yellow half of containers to find *Y *shapes, and the green half of containers to find *G* shapes, but toddlers did not. These results demonstrate that certain shape–color associations in toddlers are not the result of learned semantic associations between letter names and color names. It is controversial, however, whether stronger conclusions about innateness are warranted. First, several environmental sources of these associations have been proposed (cf. Spector and Maurer, 2011). Second, the relationship between these shape–color associations and grapheme–color synesthesia is unclear. Graphemes are not shapes *per se*, but simply the smallest contrastive unit in an orthography that is capable of distinguishing meaning. Thus the same letter can take on many different shapes and a grapheme–color synesthete will generally see all these shapes as having the same color, e.g., *A *and *a *would be seen as the same shade of crimson (for a rare report of an exception to this rule, see Ramachandran and Hubbard, 2001a), and identical shapes that are contextually determined to be different graphemes will be perceived as different colors (Dixon et al., 2006). Furthermore, for adult synesthetes graphemes induce highly specific colors (approximately as specific as color memory Arnold et al., 2012), not simply color categories or any nearby colors irrespective of color category boundaries. Thus further experimentation is required to resolve several questions. What is the precise nature of the toddler associations – what aspect of shape is associated with what aspect of color? Are these associations learned or innate? And are they the basis of the later associations between graphemes and highly specific colors in adult synesthetes?

It is important to note that despite all these first-order influences, any two synesthetes’ sets of inducer–concurrent pairings are likely to appear entirely unrelated to each other. Even the most powerful first-order effects, such as *O *being white for grapheme–color synesthetes, have many exceptions, and most of these effects are only apparent after careful statistical analyses of large populations. However, the vast majority of synesthetes we have had the opportunity to study appear to be influenced by at least some of these factors.

*The synesthetic concurrent that is associated with a particular inducer is often determined by what the synesthete has learned about that inducer or experiences they have had with the inducer*.

### SECOND-ORDER INFLUENCES ON SYNESTHETIC DEVELOPMENT

Second-order mappings can be thought of as “relations between relations.” Unlike first-order mappings, in which single elements in one domain are mapped to single elements in another domain (e.g., the letter *A *is mapped to the color red), in second-order mappings, patterns, or relations within one domain are mapped to patterns or relations within another domain. On reflection this is not surprising, given that human perception often deals with relationships rather than absolute stimulus values: for instance we can identify a tune such as Happy Birthday (a pattern of relationships between notes) better than we can individual notes.

A number of second-order mappings influence synesthetic concurrents. For instance, letters that have similar *shapes *(e.g., *E *and *F*) tend to have similar *colors *among grapheme–color synesthetes whose native language is English (Brang et al., 2011b; Watson et al., 2012a), German (Jürgens and Nikolic, 2012), or Japanese (Asano and Yokosawa, 2013). In these cases, similarity relationships within the letter–shape domain are mapped onto similarity relationships within the color domain. Second-order mappings such as these can be entirely independent of first-order mappings, and thus would not show up using first-order analyses. Thus *E *might have completely different colors for synesthetes John and Jane, but if John’s color for *E *is similar to his color for *F*, and Jane’s color for *E *is similar to her color for *F*, then the two have the same second-order mapping of shape to color.

Similarity in terms of shape is not the only type of letter similarity that affects synesthetic color. The relative frequency of letters also matters: there is a tendency for letters and numbers that are more frequently seen in print to be associated with brighter colors (Beeli et al., 2007; Cohen Kadosh et al., 2007; Smilek et al., 2007; Simner and Ward, 2008; Watson et al., 2012a), with more saturated colors (Beeli et al., 2007), and with colors whose names are more commonly spoken or written (Rich et al., 2005; Simner et al., 2005). Thus there is a second-order mapping between similarity in terms of letter/digit frequency and similarity in terms of luminance, saturation, and color word frequency.

The phonological similarity of letters also affects their synesthetic colors, although this may be a language-specific effect. Japanese *hiragana *and *katakana *characters with the same pronunciations tend to have the same colors, despite large differences in shape (Asano and Yokosawa, 2011), and hiragana characters with similar pronunciations tend to have similar colors (Asano and Yokosawa, 2013). However phonology may not be able to influence synesthetic color in opaque orthographies, where letters have multiple pronunciations. In English, for example, similarity between the sounds of letter names does not correlate with synesthetic color similarity (Watson et al., 2012a).

Another important form of letter similarity is in terms of alphabetical order, which has a somewhat curious relationship with synesthetic color: letters that are earlier in an alphabet tend to have less similar colors than later letters, at least among native speakers of English (Eagleman, 2010; Watson et al., 2012a) and Japanese (Asano and Yokosawa, 2013). The original explanation of this finding treats alphabetical order as a kind of proxy for the learning order of letters, as letters are generally learned in roughly alphabetical order (Justice et al., 2006). The idea is that as synesthetic children begin learning letters, they assign each letter a distinctive color, but as they learn more and more letters they run out of colors and are forced to use colors that are similar to those they had already assigned to previously learned letters (Eagleman, 2010; Watson et al., 2012a).

This interpretation, however, is hard to reconcile with the lengthy developmental trajectory of synesthesia. By age 7, the majority of children will have learned all their letters, but the synesthetes among them will have consistent color associations for only a third of these letters (Simner et al., 2009a), and will not develop color associations for many of them until their teenage years. It seems unlikely that these associations develop in alphabetical order, given that all the letters are well-known by this point. Thus most letter–color associations are not developed in anything like the order of learning of letters, which casts doubt on the theory that synesthetic alphabetic ordinality–color effects come from the order of acquisition of letters. An alternative possibility is that ordinal position is itself an important aspect of how alphabetic letters are conceptualized and encoded in the brain, and that any such important aspects will inevitably have an impact on synesthetic color (cf. Asano and Yokosawa, 2013).

Music–color synesthesia also displays second-order influences, although it is less clear that these influences are due to learning. Higher pitches tend to be associated with brighter colors (Marks, 1975), and quartertones tend to be associated with colors that are closer to the midpoint of the colors of the two adjacent semitones (Head, 2006). This pitch–brightness correspondence is not a unique feature of synesthesia: it is ubiquitous among non-synesthetic humans and even chimpanzees (Ludwig et al., 2011).

These second-order effects are independent of each other, at least in the case of grapheme–color synesthesia. Thus synesthetes who display a strong ordinality–color effect are no more or less likely to display a strong shape–color effect, and so forth (Watson et al., 2012a). And, as with the first-order effects, any synesthete might show no signs of a given second-order effect: these are statistical tendencies, not firm rules. Nevertheless, they can be powerful indeed, as shown by the fact that fully 79% of the variance in synesthetic color difference for Japanese hiragana is explained by a model that includes ordinality, shape, phonological, and familiarity differences (Asano and Yokosawa, 2013).

Not all second-order effects need to be learned, and several have been interpreted as arising from general developmental processes independent of experience (Maurer et al., 2012). These include mappings between visually perceived angle, height, lightness, size and auditory pitch, and sounds and shapes. One might also argue that, e.g., shape–color and sound–color similarity mappings in grapheme–color synesthesia are due to an innate tendency to map similarity to similarity. However the identification and discrimination of the individual elements in many of these mappings will require extensive learning as well (the difficulties in learning to reliably discriminate letter shapes, for instance, were discussed in the previous section), and learned contingencies could also account for some of these mappings (as is noted by Maurer et al., 2012). Furthermore, there is strong opposition from Deroy and Spence to the notion that *any *of these associations are unlearned, and several methods by which they could arise from environmental learning have been proposed (Spence and Deroy, 2012; Deroy and Spence, 2013a, b). As with the discussion of first-order relations, we do not intend to deny the possibility of unlearned influences on second-order relations, but we do suggest that learning will be an important influence on most, if not all, of them.

Finally, we note a rare example of how explicitly understood similarity relations can be used to create synesthetic mappings. In order to be able to sing in foreign languages, a problem for opera singers, Jasmin Sinha uses a synesthetic version of the international phonetic alphabet (IPA) chart of vowel phonemes. She explains how she sings vowels correctly:

When I need to identify a vowel accurately in order to sing it, I always make mental use of the (IPA) vowel chart that I first encountered as a tool while studying linguistics at university. I can assign the vowel that I need to sing to a precise location on the chart and it hardly ever coincides exactly with one of the spots that are marked. I almost always need to move the position of the desired sound on the chart a little bit.

Synesthesia does not come into it until the next stage, when I start to make conceptual use of what I learned in my linguistic studies [...] I see the layout of a vowel chart on my internal monitor, which is set up about 20–30 centimeters in front of me like a cinema screen. This screen is not flat; it curves in a wide arc in front of my face, but does not encircle my head. It looks like a large, under-exposed and grainy old black and white photograph, in which nothing can be made out except the grainy texture; the edges are rather faded. The basic color tends towards what is almost a very dark sepia; it is not simply black.

With a conscious act of will I project a horizontal vowel chart onto this screen. The chart’s bottom line is quite close to my chin and it then turns away from me with a slight lean to the left (this means that the open “a” is closest to my chin). In addition to the fact that my subjective vowel chart does not correspond exactly with the official one, I have given it an extra personal and synesthetic dimension, resulting in a three-dimensional structure. The non-synesthetic grid, which is based on the official vowel chart, forms the upper part; it “floats” above a colored layer which depicts my vowel colorings (Dittmar, 2009, p. 199).

In summary, the *relations between synesthetic concurrents often preserve many of the important learned relations between their inducers*.

### CAN SYNESTHESIA BE LEARNED BY ADULTS?

A number of studies have tried training adult non-synesthetes to make synesthetic–style associations. These studies have all employed different training paradigms, such as trial-and-error learning in which participants choose from among several colored patches when presented with a black grapheme and are given feedback on each choice (Brang et al., 2011a), having participants read novels with colored letters at their own pace (Colizoli et al., 2012), speeded visual search tasks with colored letters (Kusnir and Thut, 2012), or even post-hypnotic instructions to associate particular numbers and colors (Cohen Kadosh et al., 2009). Training times range from 15 minutes or less (Brang et al., 2011b) to 20 days (MacLeod and Dunbar, 1988), although most are well under an hour. This heterogeneity makes cross-study comparisons difficult, but there are several points worth considering.

These studies show that non-synesthetes can behave like synesthetes on the “synesthetic Stroop task.” In this task, inducers are presented in colors that are either congruent or incongruent with their associations and the subject must name the color as quickly as possible. Response times are slower when the color is incongruent with their association (Mills et al., 1999; Dixon et al., 2000). Several training studies find a similar effect among non-synesthetes with trained associations (Elias et al., 2003; Colizoli et al., 2012; experiments 2 and 3 in MacLeod and Dunbar, 1988; Meier and Rothen, 2009), although others do not (Kusnir and Thut, 2012; experiment 1 in MacLeod and Dunbar, 1988), which may be due to insufficient training time (cf. MacLeod and Dunbar, 1988).

Phenomenological reports from participants in these studies are particularly interesting, although only one study collected these in a formal manner (Colizoli et al., 2012), and several give no account of phenomenology at all (Calkins, 1894; Nunn et al., 2002; Elias et al., 2003; Brang et al., 2011b; Brang et al., 2013). Participants in 3 studies reported not having color experiences (Kelly, 1934; Meier and Rothen, 2009; Kusnir and Thut, 2012), but other studies have more promising results. Several participants in the colored novel-reading study reported that they began experiencing colors when thinking about certain letters, and the degree to which they endorsed having such experiences was correlated with the strength of their synaesthetic Stroop effects (Colizoli et al., 2012). One participant in a shape-color training study reported that “the shapes began to take on the color of the names assigned them even on training days, when they appeared in white” (MacLeod and Dunbar, 1988). Participants in the post-hypnotic suggestion study reported that numbers on license plates or street signs took on their associated colors (Cohen Kadosh et al., 2009). Finally, participants in a tone-color training study reported seeing a color when its corresponding tone was played, even if the actual visual stimulus was faintly colored with a different hue (Howells, 1944). Importantly, then, subjects in one training paradigm (Colizoli et al., 2012) report experiences that are not unlike reports made by “associator” synaesthetes, who do not experience their concurrents located in external space (Dixon et al., 2004), particularly “know-associators,” who do not experience their concurrents in a strong perceptual manner at all (Ward et al., 2007), while subjects in others (Howells, 1944; MacLeod and Dunbar, 1988; Cohen Kadosh et al., 2009) report experiences that resemble reports of “projector” synaesthesia, in which participants experience concurrents in the external world (Dixon et al., 2004), specifically the “surface-projector” variety, in which these concurrents appear on the inducers themselves (Ward et al., 2007). It is also interesting to note that three studies which had participants who reported synaesthesia-like experiences were among the longest studies (Howells, 1944; MacLeod and Dunbar, 1988; Colizoli et al., 2012), and one showed that an identical training regimen used for a shorter length of time did not lead to such reports (MacLeod and Dunbar, 1988). No doubt there are important differences between the experiences of participants in training studies and long-term synaesthetes, but the weight of the evidence from self-reports certainly seems to suggest that there are equally important similarities.

Phenomenal experiences aside, trained participants in these studies differ from developmental synesthetes. Participants in the novel-reading study had poor recall of their letter–color associations, remembering less than half of their associations 6 months after training had stopped, whereas synesthetes typically have close to perfect consistency in their reported associations over much longer periods of time (Colizoli et al., 2012). Participants who had been trained with letter–color associations and subsequently learned conditioned responses to colors did not transfer these responses to their associated letters, unlike synesthetes given the conditioning task (Meier and Rothen, 2009). Nor did participants trained with word–color associations show increased activation in visual cortex when listening to words, unlike matched synesthetes (Nunn et al., 2002). However given that the synesthetes in such studies have had decades of experience with their associations, the short time spent in training (respectively, long enough to read a single novel, 70 min, and possibly less than 10 min) is hardly sufficient to draw any strong conclusions (as is noted by Meier and Rothen, 2009). One neurophysiological study did test an individual with much more training: a cross-stitcher with 8 years of experience viewed numbers that she associated with thread colors (Elias et al., 2003). Unlike a grapheme–color synesthete performing the same task, she showed no activation of visual cortex. Note, however, that this is a single null result from a single subject. Given that only two studies have compared the neurophysiological correlates of trained and synesthetic associations (Nunn et al., 2002; Elias et al., 2003), and given that neurophysiological results in developmental synesthetes are notoriously hard to replicate (see Rouw et al., 2011 for a review), a pair of null results using different training paradigms and different types of synesthesia is impossible to interpret.

In summary, *non-synesthetic participants in a number of training studies report experiences that sound quite similar to genuine synesthetic experiences. There are important differences between the effects of short-term training in adults and the long-term associations of synesthetes, but these are not enough to conclude that the two are qualitatively distinct.*

## HOW SYNESTHESIA INFLUENCES LEARNING

Above we reviewed the studies that have examined how learning affects synesthetic experience. Influences in the other direction – from synesthetic experience to its use in learning – have received less attention. In this literature researchers have focused primarily on enhanced memory. There have been very few studies on the complex forms of learning that synesthetes commonly report their synesthesia helps with, such as learning to speak foreign languages, how to score music, to hear musical intervals and musical key changes, and to understand correct musical phrasing, or even to hear the phonemes of vowels for both native and foreign languages. “Pure” memorization is clearly necessary but not sufficient for such tasks, meaning that there is a lack of evidence about potentially important aspects of synesthesia.

In a nutshell, the studies summarized below suggest that synesthesia can enable both implicit and explicit forms of learning. That is, synesthetes can exploit their concurrents as a means of obtaining and retaining information about their inducers. In some cases they do this deliberately, even to the extent of planning or training themselves to use their concurrents in this manner, while in other cases they learn spontaneously and without apparent awareness.

### THE DIFFICULTIES OF STUDYING SYNESTHETIC LEARNING

There are a number of reasons why this area of research is under-developed. In part, the problem lies in delineating a number of specific claims and finding the appropriate experimental methods to test them. Here we list some of the complications facing experimenters in this field.

First, we need to ask whether synesthesia helps synesthetes to learn and/or organize their thoughts or whether it merely *seems *to do so. It is possible that despite their rich experiences, synesthetes use the same cognitive resources as non-synesthetes to remember their appointments throughout the week, sing the proper vowels and so on. Is their phenomenology actually connected to how synesthetes learn? Seron et al. (1992), for example, report that they were unable to determine whether their participants’ number-forms are actually used in calculation.

Second, synesthesia might provide an *alternative *way to master a skill, but not necessarily a *superior *way. Certainly the superiority claimed by many synesthetes could result from a failure to imagine how non-synesthetes get about the world without these experiences. As Melanie Ahrling remarked: “I don’t understand how anyone can orientate themselves in time without such schemata in their heads, but obviously it does work and for many people it functions just as well for them as it does for me – except without the colors and little boxes.” (Dittmar, 2009, p. 147). In this case, studies that merely look for advantages on certain tasks might be missing important differences.

Third, synesthetic styles of learning might even prove a hindrance. An example of this comes from a synesthete who reports that when she tried to learn the piano, she discovered that she had three different and inconsistent sets of colors: one for each of her fingers, one for each of the musical pitches, and another for the letter names of the musical notes. These inconsistencies proved insurmountable, and so she gave up trying to learn the piano and switched to the Theremin (Pautzke, 2010)! A recent study has also shown that grapheme–color synesthetes are impaired when trying to memorize randomly chosen associations between colors and graphemes for which they do not consciously experience synesthetic colors (Brang et al., 2013). In general, any advantages might be counterbalanced by limitations.

Finally, synesthesia often involves complex, multimodal experiences. For example, a grapheme–color synesthete might experience the letter *P *as a specific stable color. But *P *might also have any number of other properties (Eagleman and Goodale, 2009): it could emit light or glow, be composed of a mix of colors, have a certain level of specular reflectance (shiny or dull), a texture (silk or sandpaper), a personality (boring or overbearing), or a taste (salty or sour). Any or all of these properties might be relevant to the role that *P *plays in the individual’s system of learning.

A recent documentary illustrates this complexity nicely (Kirschner and Söffig, 2012). In it, a number of synesthetic members of a boys choir discuss how they use their synesthesia to “code” a heard song in order to reproduce it. For example, one boy sees each note as a colored square; volume affects the size of the square and its color represents pitch. Another boy represents the song as a series of colored dots that form a line stretching out to the left of the child; each note within a phrase receives the same color, and pitch is represented by a line of dots that dips and rises. A third represents the same song with elaborately colored, personified, hopping numbers, shapes and stick persons.

What the boys are learning, namely rote-singing, is a complex set of skills that allow one to reproduce long passages of music from a single hearing. They must learn to hear the music, to discriminate changes in pitch and discern the melodic contour; to commit the melodic phrase to memory; and finally to reproduce the melody, matching the voice to each remembered note while monitoring and correcting vocal errors. Like chess masters who perceive structure and relations between pieces in a few glances (cf. Reingold and Charness, 2005), skilled rote singers have learned to perceive musical relations rather than collections of notes. This is true even for musicians with perfect pitch and for musical savants (Halpern and Bower, 1982; Sloboda et al., 1985; Young and Nettelbeck, 1995; Miller, 1999). Children who are explicitly taught pitch relations and the melodic contours of tonal music through explicit explanations of musical concepts are able to learn melodies more quickly, understand melodic contours better, make fewer mistakes in memory, and sing more accurately, results that are in line with adult performance (Petzold, 1963; Apfelstadt, 1984; Oura and Hatano, 1988; Hedden, 2012).

One can imagine several ways in which each of the boys’ synesthesias might help with any of these skills, but confirming that this is what actually happens would be difficult indeed. Recall the boy who experienced melody as an undulating line of colored notes. An obvious interpretation is that he represents pitch contour spatially and note identity with color. Even so, his perception of pitch contour might be no better than average. He may have substituted his synesthetic representations of relational pitch for whatever representational methods are used by non-synesthetes, in much the same way that children who are taught alternative multimodal forms of representation may use those types of representation. Then again, despite his own reports, his synesthetic experiences may play no role in his memory of pitch sequences. Finally, even if his memory for pitch contour is exemplary, there is no guarantee that his singing will be more accurate than that of other choir members. Vocal production of a song requires more than accurate memory, such that relational encoding fails to enhance vocal matching. In this particular case, the choirmaster reports that the synesthetic boys are not especially skilled in comparison to the other choir members (Kirschner, personal communication).

For many reasons, then, understanding and testing how synesthesia affects learning is truly difficult, and will often require a deep understanding of the specifics of each individual synesthete’s experiences in order to develop appropriate experiments (cf. Smilek and Dixon, 2002). Despite these challenges, there has been some success in verifying that synesthesia can and does affect certain forms of learning, and we now turn to these cases.

### IS THERE A SYNESTHETIC ADVANTAGE FOR MEMORY?

Probably the single most common anecdotally reported benefit of synesthesia is that it helps with memory. This example is typical:

My most crucial guide to orientation is my colored numbers. Above all, they help me to remember telephone numbers and birthdays.

I have two uncles whose birthdays are very close together: an uncle August – whose birthday is on the 28th of November – and an uncle Berthold – who celebrates his birthday on November 30th.

Because the name August is a violet-dark blue and the name “Berthold” is a yellow–brown, I have never in my entire life got these birthdays muddled up – as my number 8 is dark-blue and my 3 is yellow. ….In this instance, the colors of the names and the colors of the birthday–dates match up so well that they serve as an excellent memory aid (Dittmar, 2009, p. 140).

Grapheme–color synesthetes often describe how their colors assist with remembering names, telephone numbers, or the spellings of words (cf. Chapter 2 in Cytowic, 2002), and they generally report above-average memory abilities (Yaro and Ward, 2007). Several recent studies have formally investigated the relationship between synesthesia and memory, using both case and group studies. Readers interested in a more detailed account of this research should consult the review by Rothen et al. (2012).

Single case studies usually focus on synesthetes with extraordinary memory abilities. These include synesthetes who explicitly claim to have exploited their synesthesia to aid them in memorizing pi to over 20,000 decimal places (Bor et al., 2007), remembering long lists of names for many weeks (Mills et al., 2006), perfectly recalling a list of random words on a surprise test 20 years after initial exposure (Luria, 1968), or perfectly recalling several 50-digit matrices several months after studying them for a few minutes (Smilek et al., 2002). In the latter case, researchers verified the synesthete’s claim that she was exploiting her synesthetic colors on this task by presenting her with matrices of numbers that were colored incongruently with her concurrents, which caused her performance to plummet well below the level of non-synesthetes (Smilek et al., 2002).

Group studies, on the other hand, have focused mainly on grapheme–color synesthesia, with one study examining time–space synesthetes (Simner et al., 2009b). In all cases, they have shown either no general memory differences between synesthetes and non-synesthetes, or synesthetic advantages that are impressive, but not near savant levels (cf. Rothen and Meier, 2010a).

What are these advantages? Synesthetes tend to have superior memories for the specific stimuli of their concurrent domain(s), e.g., grapheme–color synesthetes have advantages on various tests of color memory (Cohen’s *d* or Glass’ Δ ranging from 0.54 to 1.20; Yaro and Ward, 2007; Rothen and Meier, 2010a; Pritchard et al., 2013; Terhune et al., 2013), and calendar–form synesthetes have visual short term memory advantages (Cohen’s *d* = 0.35, Simner et al., 2009b). Furthermore, they often show memory advantages for stimuli from their inducer domain, thus grapheme–color synesthetes have advantages for various memory tasks involving lists of words (Cohen’s *d* ranging from 0.75 to 1.38; Yaro and Ward, 2007; Rothen and Meier, 2010a; Gross et al., 2011; Radvansky et al., 2011). They are also better at implicitly learning artificial grammars, but only if these grammars are composed of the letters that trigger their synesthesia (Cohen’s *d* = 0.62; Rothen et al., 2013). Calendar–form synesthetes have much better autobiographical and somewhat better historical memories (Cohen’s *d* of 1.95 and 0.87, respectively; Simner et al., 2009b).

However synesthetes show no advantages for some other memory tasks involving their inducers. Grapheme–color synesthetes do not appear to have a group advantage for remembering matrices of numbers (Yaro and Ward, 2007; Green and Goswami, 2008; Rothen and Meier, 2009), nor for retaining graphemes in working memory (Rothen and Meier, 2010a; Gross et al., 2011; Terhune et al., 2013). Furthermore they have advantages in the reproduction and recognition of simple visual figures that are not associated with their synesthetic inducers or concurrents at all (Rothen and Meier, 2010a). Thus not all synesthetic memory advantages can be tied to synesthetic experiences.

### IS THERE A SYNESTHETIC STYLE OF MEMORY?

Clearly, the research on synesthesia and memory has ambiguous results. We suggest that this is because this is a relatively new area of research and the appropriate experimental methods have not yet been determined. Most studies have concerned themselves primarily with determining whether synesthesia is associated with memory advantages, which is an interesting question in its own right, but may be irrelevant to determining whether synesthesia is exploited for memory. As discussed above, the question is not whether synesthetes perform *better *than non-synesthetes on memory tasks, but whether they perform *differently*.

What is known about such differences comes either from self-reports or from a single experimental technique that has been employed in a handful of studies: using incongruently colored stimuli in an attempt to impair synesthetes’ performance (Smilek et al., 2002; Yaro and Ward, 2007; Green and Goswami, 2008; Rothen and Meier, 2009; Radvansky et al., 2011). At present two of these studies have found no evidence of interference from incongruent colors among groups of synesthetes (Yaro and Ward, 2007; Rothen and Meier, 2009), while two group studies and one case study have found interference (Smilek et al., 2002; Green and Goswami, 2008; Radvansky et al., 2011).

One possible explanation for these inconsistencies is the choice of tasks. The stimuli used in most group studies are not ones that synesthetes have reported using their concurrents to help remember. Names have been used in one only study, which did find a strong benefit for the (single) synesthetic participant (Mills et al., 2006), and to our knowledge no one has formally tested synesthetic recall of phone numbers. Group studies where adult synesthetes were tasked with remembering large matrices of numbers showed no synesthetic advantage (Yaro and Ward, 2007; Rothen and Meier, 2009), but the synesthetes in these studies had not previously claimed to have unusual memories for such matrices. On the other hand, in every case where synesthetes have been tested *on the class of stimuli for which they actually claim to use synesthesia as a memory aid*, they have shown advantages (Luria, 1968; Smilek et al., 2002; Mills et al., 2006; Bor et al., 2007; Yaro and Ward, 2007; Simner et al., 2009b; Rothen and Meier, 2010a; Gross et al., 2011; Radvansky et al., 2011). Note that in the two cases where such advantages have been demonstrated and incongruently colored stimuli were employed to interfere with synesthetic experiences, synesthetic memory advantages were mitigated (Radvansky et al., 2011) or eliminated entirely (Smilek et al., 2002).

Clearly more research is necessary, hopefully involving novel ways of testing for specifically synesthetic styles of performance on memory tasks. Nevertheless, two key points have now been well established. *First, synesthetes tend to have memory advantages* of about 0.5–1 standard deviations over non-synesthetes for specific types of stimuli, particularly those from their inducer and concurrent domains. Second, in at least some cases, they can exploit their concurrents in the encoding or retrieval process, and this use of synesthesia as a deliberate mnemonic can be powerful indeed.

### SYNESTHESIA ENABLES NON-DECLARATIVE LEARNING

With only one exception (Rothen et al., 2013), the memory studies from the previous sections all tested explicit memory, and did so by asking participants to consciously study stimuli for later recall. One study examined whether synesthesia can also enable more implicit forms of learning, specifically classical conditioning (Meier and Rothen, 2007). Participants passively viewed a stream of slides that were either pure color patches, the colors of which changed from trial to trial, or were white with graphemes on them. During a conditioning phase, slides of one color, e.g., blue, were presented simultaneously with a startling sound. Synesthetes began displaying heightened skin conductance responses to both blue slides and white slides with graphemes that triggered blue concurrents, but not to other colors or graphemes, despite the fact that the graphemes had never been presented together with the sound. Thus conditioned responses to colors transferred to graphemes, indicating an unusual form of non-declarative learning. *Synesthetic associations, then, can be the basis of implicit learning about environmental regularities.*

### SYNESTHESIA ENABLES EXPLICIT CATEGORY LEARNING

Only one study to date has sought to verify that synesthesia can be exploited for more complex learning (Watson et al., 2012b). In this study, grapheme–color synesthetes performed a difficult category learning task where stimuli (pairs of letters) were custom-chosen for each synesthete such that the simplest rules that defined each category were based on their synesthetic colors, e.g., “a pair of red and green letters belongs in Category 1.” Crucially, each synesthete’s stimulus set contained several letters with highly similar colors. If B and R were both red, they could be used interchangeably. Synesthetes were very successful at learning this category structure, and results show that they exploited their colors to do so.

Synesthetes viewing black letters reported that they learned to consciously use their colors to categorize stimuli, though they had not been instructed to do so, and their pattern of results was consistent with this report. Specifically, they learned to categorize training stimuli accurately and after training they could correctly generalize their performance to novel stimuli that followed the same color rules. Furthermore, on a memory task in which they were asked if they had seen a stimulus before, they were unable to correctly reject novel stimuli that followed the same color rules, indicating that they had been attending to the synesthetic colors of letters, rather than to the specific letter identities. On all three tasks (training, transfer, and memory) their performance was similar to that of non-synesthetes viewing colored letters, and unlike that of non-synesthetes viewing black letters. The non-synesthetes viewing black letters were less accurate at categorizing training stimuli, unable to categorize novel stimuli correctly, and much *more *accurate at correctly rejecting novel stimuli that followed the color rules. These differences all make sense, given that they had no knowledge of the letters’ colors and could only attend to letter identity: their initial learning was poor because they could not utilize the simpler color rules, they could not use color rules to transfer to novel stimuli, and they could not be misled by memory foils that were similarly colored to stimuli they had seen before.

Again, more research is clearly needed into the use of synesthesia for more complex learning tasks. Nevertheless this single study demonstrates quite clearly that *synesthetes can consciously use their concurrents to make novel and difficult abstractions and categorizations.*

## A LEARNING HYPOTHESIS OF SYNESTHETIC DEVELOPMENT

This paper has so far consisted of a review of research on how learning influences synesthesia and how synesthesia influences learning. We have tried to present results as objectively as possible. Now, however, we switch to a completely speculative mode, and present a novel theory of synesthetic development that brings both streams of research together. As with most research on synesthesia, this theory is most fully developed in regards to grapheme–color synesthesia, but it is meant to apply to most known varieties or synesthesia, and certainly to all those varieties with explicitly learned inducers (which, as we noted previously, constitute the vast majority of known cases). We outline its relationship to other theories of synesthetic development and try to anticipate objections. An earlier version of this theory has already been published (Watson et al., 2010), and we have outlined aspects of it in discussions of our experimental work (Watson et al., 2012a, b), however this is the most complete and up-to-date presentation of these ideas.

### SYNESTHESIA DEVELOPS BECAUSE IT IS USEFUL FOR LEARNING

The theory states that synesthesia develops, at least in part, as a response to the challenges involved in learning to recognize, discriminate, and understand the relations between the members of the inducer class. That is, grapheme–color synesthesia develops because it helps the child learn various things about letters, time–space synesthesia develops as a means of learning about units of time, and so on. Similar claims were advanced well over a century ago (Galton, 1881; Calkins, 1893), but this line of thinking has not been prominent in modern research (with some exceptions, e.g., Seron et al., 1992).

Almost all synesthetic inducers, as we saw above, are category structures that are learned with much difficulty over a lengthy period of time, including rare inducers such as olfaction, taste, and audition. How might synesthesia help with this learning? We have reviewed evidence that synesthetic associations can be exploited for a variety of learning purposes. Most of this research focuses on synesthesia as a conscious memory aid or mnemonic device, and one of the initial problems that faces any child learning to use letters (or other common categories of inducers) is simply to learn to identify which letter is which. This is a far more difficult problem than is typically acknowledged, and the potential utility of a memory aid should not be underestimated. Thus for some grapheme–color synesthetes, synesthesia might arise when they are learning their letters.

However children learn a great deal more about letters than how to recognize and identify them. And while there is a paucity of research on synesthesia’s utility for other forms of learning, we have seen that synesthetic associations enable the unconscious learning of environmental regularities (Meier and Rothen, 2007; Rothen et al., 2013), and can also be consciously exploited on difficult rule-based category learning tasks (Watson et al., 2012b). In both cases, the extent and limitations of these abilities have not yet been established, but we suggest that any ability to pick up on statistical regularities of printed letters or to learn complex rules for combining letters could be highly useful at many stages during the development of literacy. More generally, such abilities could be useful when a child is faced with any of the challenges involved in learning any of the categories of synesthetic inducers.

Synesthesia in adults is generally accepted to be automatic, although there is debate over this claim (cf. Mattingley, 2009; Price and Mattingley, 2013). If it begins as a strategy, it seems unlikely that it would be straightforwardly automatic in its early stages, but with extensive rehearsal synesthetic associations could become more and more automatic as time went on, as has been demonstrated in non-synesthetes trained with synesthesia-like associations for 20 hours over the course of a month (MacLeod and Dunbar, 1988). Of course all the synesthetes tested for automaticity using Stroop or other tasks in the laboratory have had a decade or more to rehearse their associations, which would conceal any slower, strategic origins.

### MODIFYING CONCURRENTS AS A LEARNING AID

The potential utility of synesthesia as a learning strategy is magnified by the fact that synesthetic concurrents encode information about their inducers. As we saw, concurrents reflect both the first-order properties of individual inducers (e.g., the semantic associations of particular letters) and the second-order relationships between inducers (e.g., the relative positions of letters in the alphabet). Thus not only can synesthetic concurrents be a basis of learning about inducers, but they are also “chosen” and even modified on the basis of what has been learned. Given the multi-year period in which synesthetic associations are in flux, there is the potential for a lengthy period of reciprocal interactions between learning about inducers and tweaking the particular associations triggered by these inducers. This type of reciprocal relationship may enable further learning.

We have seen, however, that a wide range of inducer properties are mapped to concurrents. It is not possible for all these properties to determine the concurrents associated with each inducer, since they are not all consistent. For instance, *B *and *D *have highly similar shapes (particularly in their lowercase forms), and are also both early letters in the alphabet. Thus according to a mapping of shape similarity to color similarity, they ought to be quite close to each other, but according to a mapping in which letters earlier in the alphabet have *less *similar colors, they ought to be far apart. The semantic associations with these letters might provide a further reason for them to be far apart, as *B *might, for example, be associated with blue as its first letter, but *D *might be brown as a result of being associated with “dog,” which are stereotypically brown. Alternatively, such semantic associations might bring them close together, as *B *could instead be associated with brown as its first letter. Add into this mix their high degree of phonological similarity, differences in their frequency of usage, and the idiosyncratic personal experiences of the individual synesthete with these letters. It is obviously not possible for any two colors for *B *and *D *to simultaneously satisfy all these constraints.

What determines which constraint gets satisfied in any particular case? Asano and Yokosawa (2013) suggest that the feature making the greatest contribution to differentiating the letter from others will be the one that is most likely to have the largest influence on its color. For Japanese hiragana and katakana scripts, which are almost perfectly *orthographically transparent *(each character represents only one phoneme, and each phoneme is represented by only one character), one might expect that phonology would be a particularly important determinant of synesthetic color, while in an orthographically opaque language such as English, phonology would have far less utility.

This analysis resembles the application of optimal integration theory to multisensory perception (Ernst and Banks, 2002). This theory proposes that participants, when required to combine sensory signals from multiple sources, tend to weight the signals according to their relative reliability. Specifically, the weight of a sensory cue in a multisensory decision is directly proportional to the cue’s reliability. For example, in a study by Helbig and Ernst (2007), participants made shape judgments of elliptical objects presented simultaneously by touch and vision, but varied slightly in their shape in each modality. The results showed that when noise in the visual signal was low, decisions were dominated by vision, but as the visual noise increased, decisions were increasingly influenced by the signals received through touch. Consistent with this theory, we argue here that the utility of a synesthetic color for discriminating among letters is likely one of the factors determining its selection, but there could be many other such factors. Stated more generally, *synesthetic concurrents are likely determined by whatever aspect of the inducers is most relevant to the resolution of the learning challenge confronting the child.*

### WHAT WOULD MAKE SOMEONE MORE LIKELY TO EMPLOY SYNESTHETIC ASSOCIATIONS AS A LEARNING STRATEGY?

Recently it has become clear that synesthetes, as a group, have a number of unusual cognitive, perceptual, and personality traits. As noted previously, synesthetes have unusually strong perceptual and cognitive abilities related to their inducer and concurrent domains (e.g., Yaro and Ward, 2007; Simner et al., 2009b), but there are several other elements of this profile, including a strong connection to creativity and artistic expression (Dailey et al., 1997; Rich et al., 2005; Ward et al., 2008; Rothen and Meier, 2010b), skill at making associations between typically disparate elements (Ward et al., 2008), high rates of positive schizotypal experiences (Banissy et al., 2012), stronger verbal and vivid imagery cognitive styles (Meier and Rothen, 2013), enhanced visual imagery abilities (Barnett and Newell, 2008; Spiller and Jansari, 2008; Price, 2009), and greater openness to experience and tendency to fantasize (Banissy et al., 2013).

Consider a child who is open to novel experiences, highly skilled with visual imagery, unusually creative, skilled at making strange associations, prone to fantasizing, and possessed of a highly developed ability to discriminate and remember colors. Such a child could well be far more likely to employ an unusual visual-imagery-based strategy such as associating letters with colors when faced with the multiple challenges involved in learning to read. We suggest, then, that the “synesthetic personality” may be the personality of someone who is more likely to “stumble” into synesthesia – although of course some of this personality (which has always been measured in adults) could also be the result of living with synesthesia for many years.

Are children deliberately choosing to use synesthesia as a learning strategy, or consciously aware of doing so? It might be that such associations are spontaneously made at a relatively sub-personal or unconscious level, and that they are strengthened and maintained because they prove useful for certain tasks (cf. Galton, 1881; Calkins, 1893). But in some cases the original impetus for making these associations could be conscious deliberation about the difficult learning problem facing the child, which she will be consciously aware of in almost all cases, since, as we saw earlier, almost all synesthetic inducers are learned in a formal setting. However awareness of a learning *problem *that one has successfully solved does not always entail awareness of the *solution *one actually used, and so we remain agnostic about the degree of deliberation or consciousness involved in the strategic development of synesthetic associations.

### WHAT LINKS MULTIPLE FORMS OF SYNESTHESIA?

Different forms of synesthesia appear to be interrelated. That is, if you have one form of synesthesia you are far more likely to have other forms (Sagiv et al., 2006; Brang and Ramachandran, 2011), and there are several “clusters” of synesthetic types that are more strongly related than others (Novich et al., 2011). This interrelatedness is generally explained as stemming from a genetic predisposition to develop synesthesia that manifests itself in different forms; but we suggest another possibility, namely, that if one has successfully employed a certain learning strategy, one (or one’s brain) might be more likely to use similar strategies when faced with similar problems in the future.

### WHY ISN’T EVERYONE A SYNESTHETE?

Virtually everyone in modern industrialized societies has learned to use letters and calendars, but most do not have colors for each letter, or convoluted spatial forms for calendars. If synesthesia develops as part of a strategy for learning about such things, then why do we not see more synesthetes? There are a number of plausible answers to this question.

First, the development of synesthesia may be possible only during critical periods of development, when the systems responsible for processing and representing inducers and concurrents are plastic enough to allow such unusual connections to form. Our review of the adult training literature found some evidence that non-synesthetic adults can have experiences that resemble certain aspects of synesthesia after training for a long enough period of time. However the highly structured and long-lasting inducer–concurrent relationships of full-blown synesthesia may require early plasticity.

Second, a specific neurological profile that goes beyond mere plasticity may be required for the development of synesthesia. Numerous researchers have suggested, for instance, that synesthesia is caused by unusual connectivity between brain areas responsible for processing stimuli from the inducer domain and areas responsible for concurrent experiences. Such connectivity could take the form of structural differences, such as more axonal projections or more heavily myelinated projections between these areas (cf. Ramachandran and Hubbard, 2001b), or functional differences such as less inhibitory activation from other areas (cf. Grossenbacher and Lovelace, 2001), or some combination of these factors (Brang et al., 2010). A large number of studies have now confirmed that adult synesthetic brains differ in various ways from those of non-synesthetes (for recent reviews, see Hubbard et al., 2011; Rouw et al., 2011), including unusual connectivity in brain areas associated with inducer and concurrent representation, although there is still debate about whether these differences cause or are caused by the constant conjunction of inducers and concurrents (cf. Cohen Kadosh and Walsh, 2008).

Third, a specific genetic profile might be a prerequisite for the development of synesthesia. For instance, it is commonly suggested that the unusual connectivity associated with synesthesia stems from genetic mutation (e.g., Maurer, 1993; Ramachandran and Hubbard, 2001b), possibly to a gene (or genes) involved in the modulation of neural pruning during development (e.g., Baron-Cohen et al., 1993; Bailey and Johnson, 1997; Hubbard and Ramachandran, 2005). Until quite recently, a relatively simple genetic trigger for synesthesia seemed quite plausible. Synesthesia was thought to be rare, with rates as low as one in 2000 in the general population, yet almost 50% of first-degree relatives of synesthetes were reported to be synesthetes, representing a thousand-fold increase (Baron-Cohen et al., 1996; Barnett et al., 2008). Evidence suggesting a strong link between synesthesia and gender bolstered this genetic interpretation. The ratio of female to male synesthetes was reported to be as high as 6:1, and one well-cited study found an 8:1 ratio of female to male family members (synesthetic or not) of synesthetes (Baron-Cohen et al., 1996; Ward and Simner, 2005; Barnett et al., 2008). Moreover, in almost all reported cases of familial synesthesia, the trait was passed along the maternal line (Baron-Cohen et al., 1996; Ward and Simner, 2005; Barnett et al., 2008). Such findings were consistent with a simple (i.e., single-gene) X-linked pattern of inheritance, possibly one that was lethal to males *in utero *(Bailey and Johnson, 1997). However later research increased sample sizes and avoided several methodological flaws, and overturned most of these findings. While there is clearly a strong tendency for synesthesia to run in families, as has been known for over a century (Galton, 1883), synesthesia is far more common in the general population than was thought, with rates of grapheme–color synesthesia alone being placed at about 1% (Simner et al., 2006). Furthermore, there is no difference in the number of males and females in the families of synesthetes, ending speculation about X-linked lethality (Ward and Simner, 2005; Barnett et al., 2008). There is also likely little or no difference in the actual rates of female and male synesthesia (Simner et al., 2006, 2009a), previously reported differences likely stemming from differences in response biases between the sexes. Finally two direct genetic studies of synesthetes found multiple loci of interest for synesthetic inheritance that differed between the two studies, and were consistent with multiple modes of inheritance being involved in synesthesia (Asher et al., 2009; Tomson et al., 2011).

So there is almost certainly no simple genetic story to tell, no single gene or group of genes that “turns on” synesthesia. Still, there is clearly a genetic influence on synesthetic development, or more accurately a range of genetic influences. These could take the form of influences on neural pruning or inhibition, as is favored by many researchers, but they could equally be influences of another kind, such as a multi-factor genetic influence on the “synesthetic personality” described above. Whatever the nature of the genetic influences, they contribute to the relative rarity of synesthesia.

All neurological and genetic accounts, however, have the same shortcoming: they do not explain why almost all synesthetic inducers are explicitly taught, culturally dependent, categories (Day, 2005; Rich et al., 2005; Simner et al., 2006). If synesthesia is simply the result of a hyperconnected brain, then why do almost all the connections begin with objects of formal instruction? If grapheme–color synesthesia develops from an innate link between shapes and colors (Maurer et al., 2012), why do adult grapheme–color synesthetes not report colors for all shapes? At the very least, genetic and neurological accounts need to be able to answer these questions, and we see no way of doing so without a theory that places learning at the forefront of synesthetic development.

## CONCLUSION

We hope that this article provides a comprehensive resource for those researchers interested in the two-way influences between synesthesia and learning. With the wide range of evidence we have summarized for the influence of learning on synesthesia, we hope to have shown that far from being unlearned, “learning is the defining characteristic of synesthesia” (Witthoft and Winawer, 2013). The growing body of work on synesthesia’s utility for learning demonstrates, in addition, that it can be exploited in both implicit and explicit learning of many different kinds.

We do not expect to have convinced all our readers of the validity of our learning hypothesis of synesthetic development, but we do hope to have sparked some interest in it. We also hope that by reviewing the two-way influences between synesthesia and learning, we have contributed to the growing recognition of the importance of learning in synesthesia.

## Conflict of Interest Statement

The authors declare that the research was conducted in the absence of any commercial or financial relationships that could be construed as a potential conflict of interest.
